# Alterations in lipid metabolism gene expression and abnormal lipid accumulation in fibroblast explants from giant axonal neuropathy patients

**DOI:** 10.1186/1471-2156-8-6

**Published:** 2007-03-01

**Authors:** Conrad L Leung, Yinghua Pang, Chang Shu, Dmitry Goryunov, Ronald KH Liem

**Affiliations:** 1 Department of Pathology and Cell Biology, College of Physicians and Surgeons, Columbia University, 630 W.168^th ^Street, New York, New York 10032, USA

## Abstract

**Background:**

Giant axonal neuropathy (GAN) is a hereditary neurological disorder that affects both central and peripheral nerves. The main pathological hallmark of the disease is abnormal accumulations of intermediate filaments (IFs) in giant axons and other cell types. Mutations in the *GAN *gene, encoding gigaxonin, cause the disease. Gigaxonin is important in controlling protein degradation via the ubiquitin-proteasome system. The goal of this study was to examine global alterations in gene expression in fibroblasts derived from newly identified GAN families compared with normal cells.

**Results:**

We report the characterization of fibroblast explants obtained from two unrelated GAN patients. We identify three novel putative mutant *GAN *alleles and show aggregation of vimentin IFs in these fibroblasts. By microarray analysis, we also demonstrate that the expression of lipid metabolism genes of the GAN fibroblasts is disrupted, which may account for the abnormal accumulations of lipid droplets in these cells.

**Conclusion:**

Our findings suggest that aberrant lipid metabolism in GAN patients may contribute to the progression of the disease.

## Background

Giant Axonal Neuropathy (GAN) is a severe autosomal recessive disorder that affects both the central and peripheral nervous systems. The most prominent pathological feature of GAN is the large, focal accumulations of neuronal intermediate filaments (IFs) in distended axons [1]. Abnormal aggregations of IFs have also been found in astrocytes, endothelial cells, Schwann cells and cultured skin fibroblasts. Many GAN patients have frizzy hair that is distinctive from their parents. Chemical analysis of the hair has revealed a disruption of disulfide-bond formation in hair keratins [2]. Hence, a generalized disorganization of IFs has been proposed to be responsible for GAN [3].

Skin fibroblast explants collected from GAN patients have been used as a model to study the disease. Under normal culture conditions, a low percentage of GAN fibroblasts exhibit abnormal aggregation and bundling of vimentin IFs [4-7]. Upon various stimuli, such as low serum [5] or low doses of trypsin [6], the vimentin networks of GAN fibroblasts collapse and form aggregates. Moreover, the microtubule (MT)-depolymerizing agent nocodazole exerts different effects on normal and GAN fibroblasts. Although the IF networks of both types of fibroblasts collapse under nocodazole treatment, the aggregates formed in GAN cells are significantly more compact and dense [5]. Together, these data suggest that dysfunction of the *GAN *gene product might cause IFs to form aggregates that are harmful to cells.

A GAN gene has been identified and its product named gigaxonin, with twenty-three different mutations reported to date [8-10]. Gigaxonin is a member of the kelch repeat superfamily. It contains an N-terminal BTB/POZ (Broad-Complex, Tramtrack and Bric-a-brac/Poxvirus and Zinc-finger) domain and six C-terminal kelch motifs. MT-Associated Protein 1B (MAP1B), Tubulin Cofactor B (TBCB), and MT-Associated Protein 8 (MAP8 or MAP1S) have been identified as binding partners of gigaxonin in yeast two-hybrid screens [11-14]. Gigaxonin interacted with these proteins via the kelch repeats. The N-terminal BTB of gigaxonin could bind ubiquitin-activating enzyme E1, suggesting that gigaxonin functions as a scaffold protein in the ubiquitin-proteasome complex and mediates the degradation of MAP1B, TBCB and MAP8 [11]. Mutations in the *GAN *gene result in accumulation of these cytoskeletal proteins and eventual neurodegeneration.

Here, we report the characterization of two primary lines of cultured GAN fibroblasts carrying a total of three putative disease-linked *GAN *alleles. We compared the gene expression profiles of the GAN fibroblasts to those of normal fibroblasts. We found that the expression of lipid metabolism genes was perturbed in GAN fibroblasts most dramatically. In addition to changes in the expression levels of lipid metabolism genes, we also discovered an increase in the number of neutral lipid droplets in GAN cells. These data suggest that defects in lipid metabolism may contribute to the pathogenesis of GAN.

## Results

### Genotyping of fibroblast explants

**Figure 1:**
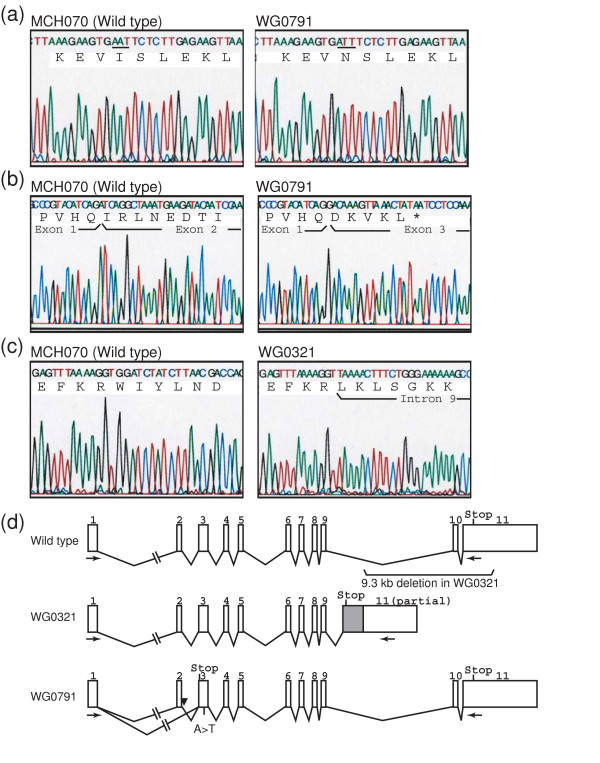
*GAN *mutations in GAN fibroblasts. (A) Sequencing of the 1.8-kb RT-PCR product from MCH070 (wild-type) and WG0791 (GAN) cells. The WG0791 product contains an A→T missense mutation. The affected codon is underlined. (B) Sequencing of the 1.7-kb RT-PCR product from WG0791 (GAN) and the 1.8-kb product from MCH070 (wild-type) cells. *GAN *exon 2 is skipped in the 1.7-kb WG0791 product, resulting in an out-of-frame premature stop codon (*). (C) Sequencing of the RT-PCR products from MCH070 (wild-type) and WG0321 (GAN) cells. The WG0321 product includes part of intron 9. (D) Schematic representation of the *GAN *mutations in WG0321 and WG0791 patients. Because of a 9.3-kb deletion in the WG0321 allele, a portion of intron 9 is included in the mRNA (gray box). The intronic mutation in WG0791 is indicated with an arrowhead. The positions of the RT-PCR primers used to amplify *GAN *cDNA are also shown (arrows).

Four subcutaneous fibroblasts explants, MCH068, MCH070, WG0321 and WG0791, were obtained from the repository for mutant human cell strains at the McGill University Health Center. MCH068 and MCH070 cells were isolated from normal individuals while WG0321 and WG0791 cells were isolated from patients diagnosed with GAN. Both GAN patients experienced difficulty in walking and their electromyography showed diffuse axonal neuropathy. We sequenced the *GAN *cDNAs prepared from the fibroblast explants. No mutations were detected in MCH068 and MCH070 cells. We obtained two PCR products from WG0791 cells, a major product of ~1.8 kb and a minor product of ~1.7 kb (data not shown). Sequencing of the 1.8-kb product revealed that a missense mutation in exon 3 (c.545T>A). The mutation resulted in the substitution of the isoleucine at amino acid position 182 with an asparagine, I182N (Fig. 1A). The 1.7-kb fragment represented an mRNA product from the other *GAN *allele because it did not contain the I182N mutation. It was shorter than the wild-type message because it did not contain exon 2 (Fig. 1B). We then sequenced the first three exons and the intron-exon junctions of the *GAN *gene from WG0791 cells. While confirming the I182N missense mutation, we also discovered an A→C mutation near the exon 2-intron 2 junction (c.282+3A>C), which might account for the misspliced message (data not shown).

Sequencing of the *GAN *cDNA prepared from WG0321 cells revealed a deletion/insertion in the *GAN *message: nucleotides 1505–2056 were replaced with a 452-nucleotide-long sequence that was identical to a part of intron 9 of the *GAN *gene (Fig. 1C). We sequenced the 3' region of the *GAN *gene from WG0321 cells and discovered that the entire exon 10 and 446 base pairs of the exon 11 5'end were deleted in both alleles. The deletion caused exon 9 to be spliced into intron 9 (data not shown). A schematic representation of the mutated and normal *GAN *alleles is shown in Fig. 1D.

Because we were unable to screen additional healthy controls, the three novel *GAN *alleles should be considered putative disease-associated mutations.

### Characterization of GAN fibroblasts

**Figure 2:**
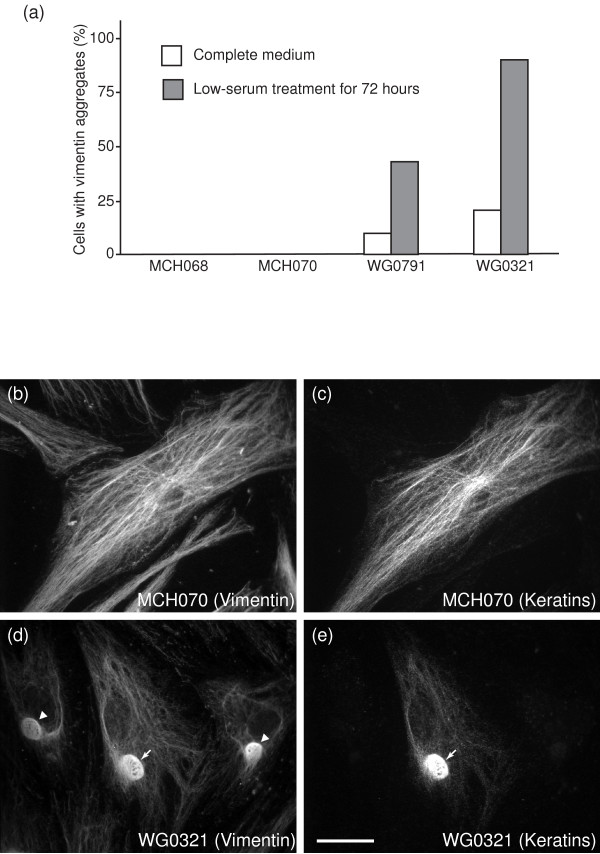
Cytological analysis of normal and GAN fibroblasts. (A) Fractions of wild-type and GAN cells containing vimentin aggregates in normal and low-serum media. Upon low-serum treatment, there was a dramatic increase of vimentin aggregates in GAN cells, from 12% to 43% for WG0791 cells and from 19% to 89% for WG0321 cells. Vimentin did not form aggregates in normal cells under any culture conditions. (B and C) Immunostaining of MCH070 (wild-type) cells with a polyclonal anti-vimentin antibody (B) and a monoclonal anti-pan-keratin antibody (C). A small percentage of MCH070 fibroblasts expressed both vimentin and keratins. Both of these intermediate filament proteins could form an extensive filament network. (D and E) Immunostaining of WG0321 (GAN) cells with a polyclonal anti-vimentin antibody (D) and a monoclonal anti-pan-keratin antibody (E). Similar to MCH070, a small percentage of WG0321 fibroblasts expressed vimentin and keratins. Both proteins could be found in the aggregates (white arrows). Note that some vimentin aggregates did not contain keratins (arrowheads in D). Scale bar, 10 μm.

Previous studies have shown that vimentin IFs form abnormal aggregates in GAN fibroblasts and that low-serum treatment enhances the aggregation. To determine whether WG0321 and WG0791 cells also contained IF aggregates, we performed immunocytochemical staining with antibodies against various IF proteins. In complete medium, 12% of WG0791 cells and 23% of WG0321 cells displayed compact vimentin aggregates. Upon low-serum treatment for 72 hours, the number of cells containing vimentin aggregates dramatically increased, up to 50% for WG0791 cells and 95% for WG0321 cells (Fig. 2A). Vimentin aggregates were never detected in normal fibroblasts, MCH068 and MCH070. Interestingly, although cytokeratins are usually not expressed in fibroblasts, a small percentage of both normal and GAN cells (~2%) exhibited positive staining for keratins (Fig. 2B–E). In GAN cells, some of the IF aggregates contained both vimentin and cytokeratin (Fig. 2D and 2E).

### Microarray analysis of GAN fibroblasts

**Figure 3:**
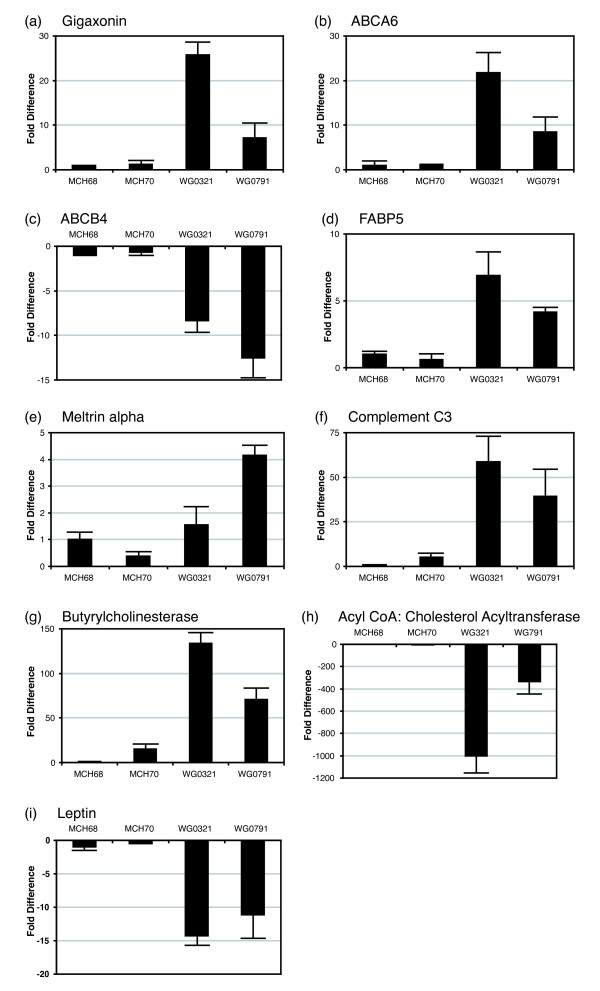
Quantitative RT-PCR analyses of the *GAN *and lipid-metabolism-related genes in fibroblast explants grown in low-serum conditions. The expression of each gene in MCH070, WG0791 and WG0321 cells was compared to that in MCH068 cells. Each data point is the mean of three separate runs. GAPDH was used for normalization.

We studied the expression profiles of GAN and normal fibroblasts grown in low-serum medium by microarray (Affymetrix) analysis. To reduce the background noise, we performed a four-way comparison of MCH068, MCH070, WG0321 and WG0791 cells. We selected genes that showed consistent changes in WG0321 and WG0791 fibroblasts when compared to MCH068 and MCH070 cells. Genes that exhibited more than three-fold differences are grouped in Table 1 in the Supplemental Data according to their proposed functions. Gene products involved in lipid metabolism displayed the most dramatic changes. We confirmed the relative expression levels of these lipid metabolism genes by quantitative RT-PCR (Fig. 3). The results were in agreement with the microarray experiments. We also performed quantitative RT-PCR to determine the expression levels of gigaxonin in GAN fibroblasts (Fig. 3A). Compared to MCH068 and MCH070, *GAN *mRNA expression was dramatically upregulated in WG0321 (~26 fold) and WG0791 (~7 fold) cells.

**Table 1:** Differentially expressed genes in GAN vs. normal fibroblasts as analyzed by oligonucleotide microarrays. Genes selected displayed at least a three-fold difference in expression level.

**Name**	**Acc. number**	**Fold change**
**Genes involved in lipid metabolism and adipogensis**		
Complement component 3 precursor	NM_000064.1	33.67
Butyrylcholinesterase	NM_000055	20.67
Alpha-2,8-sialyltransferase	L32867.1	6.95
Fatty acid binding protein 5	NM_001444.1	6.48
ATP-binding cassette A6	AA099357	4.38
Meltrin alpha	W46291	5.51
Adipsin	NM_001928.1	-7.39
ATP-binding cassette B4	NM_000443.2	-9.77
Acyl coenzyme A:cholesterol acyltransferase	S73751.1	-24.91
Leptin	NM_000230.1	-34.35
		
Integral membrane proteins and receptors		
GABA-B receptor	AF056085.1	8.85
Integrin, beta 3	M35999.1	4.89
GABA-B receptor R2	AF095784.1	5.05
GABA-B receptor splice variant 1	AF095723.1	6.06
Orphan G protein-coupled receptor	AF069755.1	4.52
Integral membrane serine protease	U76833.1	4.63
Hyaluronan-mediated motility receptor	NM_012485.1	3.14
Death receptor 6	BE568134	-4.14
Endothelin receptor	M74921.1	-5.12
P-glycoprotein (mdr1)	AF016535.1	-7.95
Membrane glycoprotein M6	D49958.1	-5.82
C18ORF1	NM_004338.1	-4.19
Glycoprotein M6A	BF939489	-6.68
Potassium channel beta subunit	L39833.1	-5.44
Endothelin receptor type B	NM_003991.1	-8.89
Potassium channel beta 1a subunit	U33428.1	-3.99
E-cadherin	NM_004360.1	-7.04
		
Cell division/proliferation/apoptosis		
Survivin	NM_001168.1	6.37
PDZ-binding kinase	NM_018492.1	4.38
Survivin-beta	AB028869.1	6.53
Mitosin (CENPF)	NM_005196.1	3.79
Dickkopf homolog 1	NM_012242.1	4.59
Kinetochore associated 2	NM_006101.1	3.51
Cell division cycle 2	AL524035	3.47
WNT4	NM_030761.1	-4.05
Fritz	U91903.1	-4.83
Tumor necrosis factor-related protein	NM_030945.1	-6.03
EGF-like-domain, multiple 6	NM_015507.2	-8.22
		
Transcription factors and nuclear proteins		
Transcription factor AP-2 alpha	BF343007	7.41
High mobility group AT-hook 1	NM_002131.1	5.58
Nuclear factor IB	AI700518	4.49
Interferon-inducible protein p78	NM_002462.2	7.57
		
Cytoskeleton		
Rabkinesin6	NM_005733.1	4.66
Stathmin-like 2	NM_007029.1	-5.32
		
Pregnancy specific beta-1-glycoprotein		
Pregnancy specific beta-1-glycoprotein 7	NM_002783.1	-5.85
Pregnancy specific beta-1-glycoprotein 4	NM_002780.1	-17.82
Pregnancy specific beta-1-glycoprotein 1	NM_006905.1	-62.90
		
Uncharacterized genes		
Hypothetical protein PRO02730	AL137654	10.56
Uncharacterized bone marrow protein	NM_018454.1	5.06
Hypothetical protein FLJ10517	NM_018123.1	5.74
KIAA0101 gene product	NM_014736.1	5.10
KIAA0008 gene product	NM_014750.1	4.62
Doublecortin and CaM kinase-like 1	NM_004734.1	4.53
KIAA1547 gene product	AW873621	3.96
Hypothetical protein DKFZp762E1312	NM_018410.1	4.54
Hypothetical protein FLJ10829	NM_018234.1	5.12
Clone HQ0310 PRO0310p1	NM_016359.1	4.38
Hypothetical protein DKFZp564H1916	AI186739	3.69
KIAA0042 gene product	NM_014875.1	4.96
Hypothetical protein FLJ22009	NM_024745.1	4.11
Hypothetical protein FLJ23468	NM_024629.1	3.48
Hypothetical protein DKFZp564N1116	BF344237	4.03
Hypothetical protein FLJ10781	NM_018215.1	-3.68
Myomegalin	AB042557.1	-4.97
Hypothetical protein DKFZp564B052	NM_030820.1	-5.46
KIAA0008 gene product	AK002054.1	-16.44
MGC:3052	BC002449.1	-7.73
KIAA0865 gene product	AI522028	-7.70
		
Miscellaneous		
Matrix metalloproteinase 1	NM_002421.2	7.73
Carbonic anhydrase XII	NM_001218.2	7.80
Topoisomerase II alpha	AU159942	6.87
Step II splicing factor SLU7	AV733266	6.93
Monocyte chemotactic protein	S69738.1	4.37
Ribonucleotide reductase M2	BE966236	4.59
Plasminogen activator, urokinase	NM_002658.1	4.19
Cathepsin C	NM_001814.1	4.34
Atrophin-1 interacting protein 1	NM_012301.1	-3.89
Monoamine oxidase A	AA923354	-4.77
Type II iodothyronine deiodinase	U53506.1	-2.80
Phosphatidylserine-binding protein	NM_004657.1	-3.98
Scrapie responsive protein 1	NM_007281.1	-5.72
Natural killer cell transcript 4	NM_004221.1	-5.94
CD24 signal transducer	L33930	-9.16
Elastase 2	NM_001972.1	-9.71
CD24 antigen	AA761181	-19.19

We also detected significant changes in members of the ATP-Binding Cassette (ABC) protein family, ABCA6 and ABCB4. ABC transporters are multispan transmembrane proteins that translocate a variety of substrates. ABCA6 has been suggested to play an important role in lipid homeostasis [15]; it was up-regulated in GAN fibroblasts. ABCB4 is also known as multidrug resistance P-glycoprotein 3 and functions as a translocator of phospholipids. Deficiencies in ABCB4 cause progressive intrahepatic cholestasis type III [16]. ABCB4 was downregulated in GAN fibroblasts. In addition, Fatty Acid Binding Protein 5 (FABP5), Meltrin alpha, Complement C3 and Butyrylcholinesterase (BChE) were upregulated in GAN fibroblasts. FABP5 is involved in intracellular fatty-acid trafficking (reviewed in [17]). Meltrin alpha is a member of the metalloprotease-disintegrin family and is involved in adipogenesis [18]. C3 is a component of the complement system of innate immunity. It is also the precursor of an acylation-stimulating protein that can increase triglyceride synthesis (reviewed in [19]). BChE is a serine hydrolase that exhibits increased activity in hyperlipidaemic patients [20]. Acyl-CoA: Cholesterol Acyltransferase (ACAT) and Leptin were downregulated in GAN fibroblasts. ACAT is an enzyme that converts intracellular cholesterol into cholesteryl esters and promotes the storage of excess cholesterol in the form of cholesterol ester droplets [21]. Leptin is a peptide hormone produced predominantly by white adipose cells; however, it is also expressed in non-adipocytes and is important in regulating fatty acid metabolism (reviewed in [22]).

### Cellular studies of lipid droplets in GAN fibroblasts

**Figure 4:**
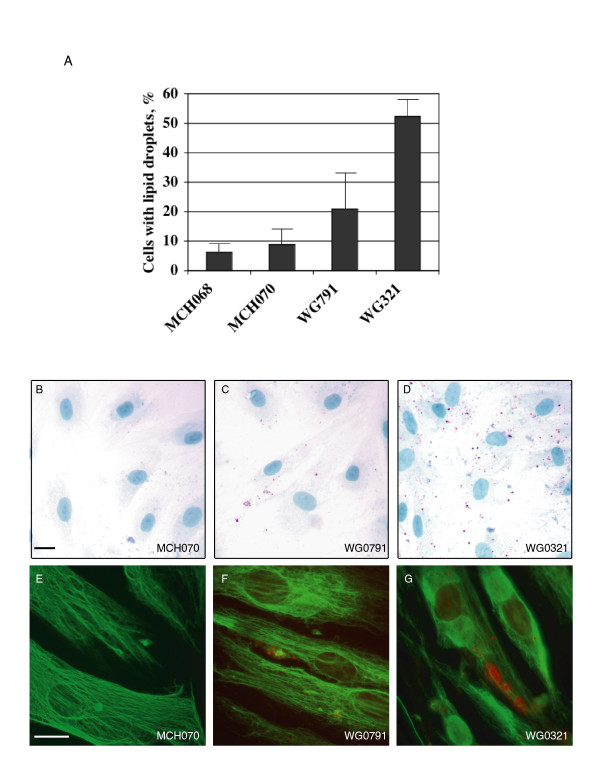
Cytological staining of normal and GAN fibroblasts grown under low-serum conditions. (A) Percentage of cells containing Red-Oil-O-positive droplets. (B-D) Fibroblasts MCH070 (B), WG0791 (C) and WG0321 (D) were stained with Oil Red O and Hematoxylin dyes. Lipid droplets were stained in red and nuclei were stained in blue. Lipid droplets accumulated in WG0791 and WG0321 cells but not in MCH070 cells. (E-G) Fibroblasts MCH070 (E), WG0791 (F) and WG0321 (G) were immunostained with monoclonal anti-vimentin V9 antibody and Oil Red O. Vimentin filaments were stained in green and lipid droplets were stained in red. Scale bars, 10 μm.

Because the microarray analysis revealed alterations in the expression of lipid metabolism genes in GAN fibroblasts, we studied the distribution of lipid droplets in the fibroblast explants by cytological staining. We used Oil Red O dye to label neutral lipid droplets of serum-starved GAN and normal fibroblasts. The proportions of cells containing lipid droplets were significantly higher in the mutant explants (52% in WG0321, 21% in WG0791) compared with the normal explants (6% in MCH068, 9% in MCH070; Fig 4A). In addition, GAN fibroblasts contained many more droplets per cell than did normal fibroblasts (Fig. 4B–D). These results were confirmed by Bodipy staining (data not shown).

Previously, it has been shown that vimentin can form cage-like structures around lipid droplets in adipocytes. We wondered whether the vimentin aggregates also surrounded the lipid droplets in GAN cells. We performed fluorescence microscopy on GAN cells co-stained for vimentin and lipid droplets. As shown in Fig. 4F–G, while some of the small vimentin aggregates appeared to encage lipid droplets, most of them did not (~80% in both cell lines).

## Discussion

In this study, we describe two GAN fibroblast explants and identify the underlying mutations in the *GAN *gene. WG0791 cells contained two different *GAN *mutant alleles, an intronic mutation near the splice donor site of intron 2 and a missense mutation in exon 3 (I182N). WG0321 cells carried two identical deletion alleles predicted to produce a truncated gigaxonin protein. As revealed by immunocytochemical analysis, both WG0791 and WG0321 cells displayed abnormal vimentin filament aggregation, a phenomenon exacerbated drastically by low-serum treatment. By comparing the expression profiles of these GAN fibroblasts to two normal fibroblasts under low-serum conditions, we found that the GAN cells exhibited defects in lipid metabolism. Unlike normal fibroblasts, which were virtually devoid of lipid droplets under these conditions, GAN fibroblasts accumulated a large number of lipid droplets.

The mechanism that caused lipid defects in GAN fibroblasts is not clear but may involve defects in the vimentin IFs. GAN has long been considered a disease of IFs, and earlier studies have shown that vimentin IFs are closely associated with cytoplasmic lipid droplets in normal cells (reviewed in [23]). Association of vimentin IFs with lipid droplets is most obvious in adipose cells where the vimentin network forms a cage-like structure surrounding the lipid droplets [24]. Similar interactions of vimentin and lipid droplets have also been observed in steroidogenic cells [25-27], and the interaction is probably direct as indicated by *in vitro *experiments [28,29]. Although the significance of the vimentin-lipid interactions to cellular functions has not been clearly defined, there is evidence to suggest that vimentin IFs play an important role in cholesterol transport. Using human adrenal carcinoma cells with or without vimentin IFs, Sarria et al. showed that there is a direct correlation between the presence of vimentin IFs and the capacity of the cells to utilize lysosomal cholesterol (Sarria et al., 1992). Their studies also indicated that the intracellular movement of low-density-lipoprotein (LDL)-derived cholesterol from the lysosomes to the site of esterification is dependent on vimentin.

Using 3T3-L1 preadipocytes as a model of adipogenesis, vimentin IFs have been shown to be important for lipid droplet accumulation during adipose development [30]. Perturbation of the vimentin network in 3T3-L1 cells during adipose conversion by nocodazole treatment, anti-IF antibody microinjection, or over-expression of a dominant-negative vimentin mutant protein could abolish the formation of lipid storage droplets in the differentiated adipocytes. The impairment appeared to be the result of an increased turnover rate of triglyceride synthesis. However, the significance of vimentin in adipogenesis has been questioned by the studies of vimentin knockout mice. Vimentin-null mice were viable and exhibited no obvious abnormality in adipose development [31]. Importantly, there was no compensatory increase in the expression of another IF protein. Only some minor pathologies were observed in the null mice. Specifically, the glial fibrillary acidic protein network was disrupted in a subset of astrocytes [32], and the Bergmann fibers of the cerebellar cortex were hypertrophic [33]. Nonetheless, cultured embryonic fibroblasts from vimentin-null mice displayed a significant decrease in the synthesis of glycosphingolipids [34]. The defect appeared to result from impaired intracellular transport of glycolipids and sphingoid bases between the endosomal/lysosomal pathway and the Golgi apparatus and the endoplasmic reticulum. It is therefore possible that, in GAN fibroblasts, mutations of the *GAN *gene affect the properties of vimentin IFs, leading to perturbation of lipid metabolism and accumulation of lipid droplets. Our observation that some low-serum-treated GAN cells contained vimentin aggregates but no lipid droplets may be explained by an insufficient sensitivity of Oil Red O staining. Alternatively, the cells could still have been in the process of accumulating oil droplets.

How do *GAN *mutations lead to defects in IF networks? One possible mechanism is through the disruption of MTs, because gigaxonin can affect the degradation of MAP1B, MAP8 and TBCB [11,12,14]. IFs are closely associated with MTs. Disruption of the MT network by nocodazole can cause IFs to collapse into the perinuclear region, and this MT-mediated effect of IFs is more obvious in GAN fibroblasts than in normal fibroblasts [5]. *GAN *mutations may therefore affect MTs, leading to IF aggregation and ultimately retention of lipid droplets. However, MT disruption with nocodazole did not have an obvious effect on the number of lipid droplets in either mutant or normal fibroblasts (data not shown). These data suggest that IF aggregation may not be linked mechanistically to lipid droplet accumulation in GAN cells, but rather that the observed defects of lipid metabolism in GAN cells are a compound effect of IF/MT perturbation and other gigaxonin-related functions.

## Conclusion

Here we describe three novel mutant *GAN *alleles, including a missense mutation, an intronic mutation, and a 9.3-kb deletion. We also characterize two GAN fibroblast explants and detect perturbations of lipid metabolism in both of them. Based on the previous studies of vimentin IFs and lipid metabolism, we speculate that the abnormal accumulation of lipid droplets that we observe in GAN fibroblasts is an indirect effect of the *GAN *mutations and is probably mediated through IF network disruption. These fibroblast explants will be a useful tool to study the physiological functions of gigaxonin.

## Methods

### Genotyping of fibroblasts

Total RNA was isolated from fibroblasts using Trizol reagent (Invitrogen). First-strand cDNA was synthesized with oligo-dT primers and reverse transcriptase (Invitrogen). The procedures were performed according to the manufacturer's protocol. Gigaxonin cDNAs were amplified from the cDNA pool using forward primer, 5'-TTGATGGCTGAGGGCAGTGCCGTGTCTG-3' and reverse primer, 5'-TTCCTCCTCAAGGGGAATGAACACGAAT-3'. After electrophoresis, PCR products were purified by the GeneClean purification system (Q-Biogen) and were sequenced with the BigDye™ sequencing kit (Applied Biosystems). The shorter gigaxonin cDNA products from explant WG0791 were first cloned into pCR2.1-TOPO vector (Invitrogen) before sequencing. The conditions used for genomic PCR and the sequences of the PCR primers have been reported elsewhere [10].

### Cell culture, immunocytochemistry and Oil-Red O staining

Fibroblast explants were obtained from the repository for mutant human cell strains at McGill University. They were maintained at 37°C and 5% CO_2 _in MEM Eagle medium (Earle's) with 10% fetal bovine serum. For low-serum treatment, cells were incubated in medium containing 0.1% fetal bovine serum for 72 hours. All experiments were performed on cells from passages 13–18. For immunocytochemical analyses, cells seeded on coverslips were fixed with 4% paraformaldehyde for 20 minutes and permeabilized with 0.1% Triton-X100 for 5 minutes. Fixed cells were incubated with primary antibodies at room temperature for one hour, followed by several washes with PBS and incubation with appropriate secondary antibodies for 30 minutes. The coverslips were then washed with PBS and mounted onto slides with Aquamount (Lerner Laboratories) for immunofluorescent microscopy. To co-stain lipid droplets and vimentin filaments, formaldehyde-fixed cells were permeabilized with 0.05% Saponin and incubated with an anti-vimentin antibody overnight. Before mounting onto slides, cells were stained for lipid droplets with 0.5% Oil Red O in propylene glycol (Poly Scientific). To stain the nuclei and the lipid droplets, cells were fixed in 4% paraformaldehyde and incubated with 0.5% Oil Red O in propylene glycol and Gill's Hematoxylin I (Poly Scientific) for 20 minutes. Antibodies used: monoclonal mouse anti-vimentin, clone V9 (Sigma), monoclonal mouse pan anti-keratin (Sigma), and polyclonal anti-vimentin [35].

### Microarray analysis

Human 133A Genechips from Affymetrix were used to study the expression profiles. Biotin-labeled cRNA probes were prepared according to the manufacturer's protocol. In brief, cells were treated with low-serum medium for 72 hours, and total RNA was extracted. First-strand cDNAs were synthesized with oligo-dT-T7 primers and reverse transcriptase (Invitrogen). After second-strand cDNA synthesis, double-stranded cDNAs were used to produce biotin-labeled cRNA probes by T7 polymerase (Enzo Laboratory). The cRNAs were fragmented before being used for hybridization. Hybridization and scanning were carried out at the GeneChip analysis facility at Columbia University.

GeneChip results were analyzed by Affymetrix Microarray Suite (version 5.0). To reduce background noise, a four-way comparison of MCH068, MCH070, WG0321 and WG0791 cells was performed. Only genes that showed consistent changes in both WG0321 and WG0791 fibroblasts when compared to both MCH068 and MCH070 cells were selected. Genes that exhibited more than three-fold differences were considered as significantly altered.

### Quantitative PCR

To determine the relative expression levels of a gene, quantitative PCR was performed on cDNAs prepared from normal and GAN fibroblasts. Single-stranded cDNAs were generated from total RNA with Oligo-dT primers and reverse transcriptase (Invitrogen). Quantitative PCR was performed on a SmartCycler II PCR machine (Cephid) with gene-specific primers, SyBr Green fluorescence dye, and OmniMix HS PCR master mix (TakaRa). The sequences of the PCR primers and the PCR conditions are shown in Table 2. GAPDH was used as the internal control, and the fold difference of a gene-of-interest in MCH070, WG0321 and WG0791 relative to MCH068 was calculated using the ΔΔCt method [36].

**Table 2:** Quantitative RT-PCR conditions and gene-specific primer sequences.

**Gene**	**Quantitative PCR Primers**	**Annealing temp.**	**Reading temp.**
Gigaxonin	F: catcgtgactgttggtggag R: tggcaatgctgtccatgtat	62°C	83°C
Complement C3	F: ctggagcagtcaaggtctacg R: gctcaatggccatgatgtact	66°C	84°C
Butyrylcholinesterase	F: aacaccgatcctccaaacttc R: tcgacattgttgagcacgtag	66°C	80°C
Sialyltransferase	F: atacctcactccagcccactt R: accatgtgctttaccaacagc	66°C	80°C
Fatty acid binding protein 5 (FABP5)	F: agttcagcagctggaaggaag R: tgaaccaatgcaccatctgta	68°C	83°C
ATP-binding cassette A6 (ABCA6)	F: actcaccgtgaaggaaaacct R: aagaccccaccttcttttcaa	66°C	84°C
Meltrin alpha	F: agtcaactcagcgagtgcttc R: ggcacttggtgtggatattgt	66°C	86°C
ATP-binding cassette B4 (ABCB4)	F: ctcgatggtcaagaagcaaag R: ttttgacctcctgagagctga	66°C	84°C
Acyl coenzyme A: cholesterol acyltransferase	F: ctgattccagaagccactgag R: ctcttctgaggcaccctcttt	66°C	85°C
Leptin	F: ccaaaaccctcatcaagacaa R: gctcttagagaaggccagcac	66°C	86°C

## Authors' contributions

C.L. – conceived of the study; performed the cell transfections and immunohistochemsitry; supervised the entire project; drafted the manuscript.

Y.P. – performed the microarray analyses; participated in experimental design.

S.C. – performed the quantitative RT-PCR; participated in data analysis.

D.G. – drafted the manuscript; participated in data analysis and sequence alignment.

R.L. – designed the experimental strategy; carried out data analysis; finalized the manuscript.

All authors have read and approved of the final version of this manuscript.
